# Local Community View of Aesthetic Surgery: Results of a Cross-Sectional Survey

**DOI:** 10.7759/cureus.33078

**Published:** 2022-12-29

**Authors:** Tareq Alyahya, Ossama M Zakaria, Abdullah AlAlwan, Maitha AlMaghlouth, Hussam Alkhars, Maisa AlAlwan

**Affiliations:** 1 Plastic Surgery, King Faisal University, AlAhsa, SAU; 2 Pediatric Surgery, Suez Canal University, Ismailia, EGY; 3 Pediatric Surgery, King Faisal University, AlAhsa, SAU; 4 Surgery, King Faisal University, AlAhsa, SAU; 5 Plastic and Reconstructive Surgery, King Faisal University, AlAhsa, SAU; 6 Plastic Surgery, King Fahad Hospital, Al Hofuf, SAU

**Keywords:** saudi arabia, community, attitude, perception, aesthetic surgery

## Abstract

Background

Aesthetic surgery has increased in popularity, reflecting the increased consumer demand. However, the variation in patients' ethnic and cultural beliefs has led to many challenges. Therefore, those who manage aesthetics should always listen and recognize the variability of cultural identities, desires, attitudes, anxieties, and uncertainties of the patient. Emerging from a diversity of cultures and its transforming trends, the scope of cosmetic surgery and its practice reflect not only the individual’s personality but also the culture as a whole. When counseling an individual, one has to recognize that even in groups of seemingly identical social or cultural standards, there are subtle differences in attitude.

Aim

To assess the perception of the local community about aesthetic procedures and to determine the possible factors influencing their level of acceptance through a randomized cross-sectional survey.

Methodology

A community-based, qualitative, cross-sectional study was performed through an anonymous questionnaire that was randomly distributed among the local population. Questions with regard to the sociodemographic data were implemented, as well as the core questions, to assess the perceptions that are based on the modified Acceptance of Cosmetic Surgery Scale (ACSS).

Results

A total of 857 participants responded to the study questionnaire. Their age ranged from 18 to more than 55 years, with a mean of 23.1 ± 12.9 years. Out of the total number, 630 (73.5%) were females while the remaining 227 (26.5%) were males. More than half of them were single (53.4%), and the remaining were married. Regarding perception, the highest score in percentage was for the interpersonal subscale (18.7 ± 7.9; 53.4%), followed by the consider subscale (18.2 7.2; 52%) and the social subscale (15.5 ± 7.9; 44.3%). The overall mean score was (52.4 ± 21.1; 49.9%). As for the procedure, the most intended was rhinoplasty (41.1%), followed by liposuction (32.9%), abdominoplasty (31.1%), face-lift (24.4%), reconstructive surgeries (24.4%), and lips filler (20.8%) while the least intended was gluteal flat grafting (8.7%).

Conclusions

Female patients are more eager to undergo cosmetic and aesthetic surgery compared to their male counterparts. Age did not have a major impact as a motive to look for cosmetic surgery.

## Introduction

Plastic surgery is distinguished as a surgical specialty for being vastly diverse. It has a wide extent of work, including both reconstructive and cosmetic procedures. The field of plastic surgery is concerned with both appearance and functional enhancements of prenatal and acquired deformities of various body parts [1]. As an evolving specialty, the work of plastic surgeons has expanded to include a wide range of sophisticated procedures that were managed by other surgical specialties in the past. However, in the past few years, there has been a substantial focus by the media on the cosmetic field of plastic surgery [1-2]. For instance, the number of cosmetic surgeries has increased significantly over the past 10 years [3].

Aesthetic surgery is defined as surgical procedures that aim to enhance appearance by improving physical features [4]. The American Society for Aesthetic Plastic Surgery (ASAPS) stated that approximately 15.6 million cosmetic surgical and minimally invasive procedures were performed in 2020. Over the past decades, an increase of 131% in cosmetic procedures was noted from 2000 to 2020 according to the American Society of Plastic Surgeons (ASPS). Furthermore, the total surgical procedures accounted for a 22% increase while minimally invasive procedures increased by 174% [5]. Despite the assumed thought that cosmetic surgeries are usually done by people who want to go back in time, patients who undergo cosmetic surgeries can be from different age groups [6].

The radical growth in the number of cosmetic surgeries recently can be attributed to three main factors. These are mainly media impact, medical advances, and patient characteristics. For the past few years, mass media has determined a highly competitive standard for beauty as the key element in determining self-image satisfaction [7-8]. It is clear that the self-image of an individual, which is evaluated to a significant extent through appearance, also affects perceptions of social roles in the world [9-10].

Regardless of the magnificent advances in the field of plastic surgery, the understanding of aesthetic surgery among the general public remains insufficient [11]. In Saudi Arabia, cosmetic surgeries were controversial in the community not only due to the health risks but also because of religious aspects [12-13]. However, some studies showed that recent advances and innovations have led to some improvements in the general attitude [14].

The current study aimed to assess the local general perception of aesthetic surgery. It’s also directed to report the possible sociodemographic factors that may influence the local population's acceptance of aesthetic surgery in a comprehensive manner.

## Materials and methods

Study design

This community-based cross-sectional study included participants from all regions of Saudi Arabia and was conducted from December 2021 to June 2022.

Population and sample size

The study targeted the Saudi population. The required sample size was 385 and was calculated using an online Raosoft sample size calculator (http://www.raosoft.com/samplesize.html) with a confidence interval of 95% at a response rate of 50% and a marginal error of 5%. Participants who were not living in Saudi Arabia were excluded from the study.

Selection criteria

These included Saudi inhabitants who were living currently in Saudi Arabia and of both genders.

Exclusion criteria

Those patients not from or in Saudi Arabia and those who did not agree to participate were excluded.

Data collection

A questionnaire was utilized to collect data from participants regarding their demographics, socioeconomic status, educational level, and perception and attitude toward cosmetic surgery. Questions were obtained from a validated Arabic version of the Acceptance of Cosmetic Surgery Scale (ACSS) that was modified for Arabic speakers by Morait et al. in Saudi Arabia with a Cronbach alpha value of 0.912 [14-15]. Permission for the reuse of the questionnaire was taken. The survey was delivered in Arabic, the primary language of the targeted population.

ACSS was developed by Henderson-King and Henderson-King and has three subscales [15]. The first is intrapersonal, which assesses whether an individual would have cosmetic surgery for self-oriented benefits. The second is social, which measures whether an individual would undergo cosmetic surgery for social reasons. The third is consider, which assesses whether an individual would consider undergoing surgery for general reasons under various scenarios. Responses were reported on a seven-point scale (1 = strongly disagree, 7 = strongly agree), with higher scores indicative of a greater endorsement of cosmetic surgery. Subscale scores were computed by taking the mean value for items associated with each subscale. The total score referred to as acceptance was computed by calculating the mean across all items. The survey we conceived was uploaded online on Google Forms. Following that, it was made available to the general public via online channels including Twitter, Telegram, and WhatsApp. To obtain a larger sample size that fairly represents all of Saudi Arabia, data collectors were used.

Statistical analysis

After data were extracted, they were revised, coded, and fed to the statistical software IBM Statistical Package for Social Sciences version 22 (IBM Corp., Armonk, NY). All statistical analysis was done using two-tailed tests. A p-value of less than 0.05 was statistically significant. As for the cosmetic surgery acceptance scale, the overall score was obtained by summing up all discrete items scores after reversing the negative sentences score. Descriptive analysis based on frequency and percent distribution was done for all variables including participants’ socio-demographic data, region, and monthly income. While the history of getting cosmetic surgery and the type of procedure participants would like to have were graphed, participants' acceptance of cosmetic surgery was illustrated in tables. The mean score with standard deviation and range was calculated for each subscale of participants’ acceptance and perception of cosmetic surgery. The relation between the participant's overall acceptance score with their personal data was tested using the independent samples t-test and one-way analysis of variance (ANOVA).

## Results

A total of 857 participants completed the study questionnaire. A total of 285 were from the Eastern region, 193 were from the Western region, 169 were from the Southern region, 119 were from the Central region, and 91 were from the Northern region. Participants' ages ranged from 18 to more than 55 years with a mean age of 23.1 ± 12.9 years. A total of 630 (73.5%) were females, 458 (53.4%) were single, and 365 (42.6%) were married. As for education, 732 (85.4%) were university graduates and 105 (12.3%) had below the secondary level of education. A total of 341 (39.8%) were employees, and 393 (45.9%) were students. The monthly income of 204 (23.8%) was less than 5000 SR while 137 (16%) had a monthly income exceeding 20000 SR (Table 1).

**Table 1:** Socio-demographic data of study participants of Saudi people

Socio-demographic data	No	%
Region		
Central Region	119	13.9%
Eastern Region	285	33.3%
Northern Region	91	10.6%
Southern Region	169	19.7%
Western Region	193	22.5%
Age in years		
< 19	55	6.4%
19-25	368	42.9%
26-35	193	22.5%
36-45	136	15.9%
46-55	77	9.0%
> 55	28	3.3%
Gender		
Male	227	26.5%
Female	630	73.5%
Marital status		
Single	458	53.4%
Married	365	42.6%
Divorced / widow	34	4.0%
Education		
Below secondary	105	12.3%
Diplomat	20	2.3%
University / above	732	85.4%
Job		
Not working	123	14.4%
Student	393	45.9%
Employee	341	39.8%
Monthly income		
< 5000 SR	204	23.8%
5000-10000 SR	258	30.1%
10000-20000 SR	258	30.1%
> 20000 SR	137	16.0%

Acceptance of cosmetic surgery among study participants in Saudi Arabia is demonstrated in Table 2. An exact 55.5% agreed that they would never have any kind of plastic surgery, 42.1% thought that cosmetic surgery is a good thing because it can help people feel better about themselves, 42% thought that cosmetic surgery can be a big benefit to people's self-image, 33% had sometimes thought about having cosmetic surgery, 32.3% said that if they knew there would be no negative side effects or pain, they would like to try cosmetic surgery, 31.6% reported that in the future, they could end up having some kind of cosmetic surgery, and 31% said that if cosmetic surgery could make someone happier with the way they look, they should try it. Only 24.3% would seriously consider having cosmetic surgery if they thought that their partner would find them more attractive, and 25% would think about having cosmetic surgery in order to keep looking young.

**Table 2:** Acceptance of cosmetic surgery among study participants in Saudi Arabia

Acceptance of cosmetic surgery	Disagree a lot		Disagree somewhat		Disagree a little		Neutral		Agree a little		Agree somewhat			Agree a lot		
	No	%	No	%	No	%	No	%	No	%	No	%	No		%	
It makes sense to have minor cosmetic surgery rather than spending years feeling bad about the way you look	101	11.8%	184	21.5%	148	17.3%	130	15.2%	101	11.8%	106	12.4%	87		10.2%	
Cosmetic surgery is a good thing because it can help people feel better about themselves	66	7.7%	146	17.0%	176	20.5%	108	12.6%	133	15.5%	118	13.8%	110		12.8%	
In the future, I could end up having some kind of cosmetic surgery	150	17.5%	183	21.4%	151	17.6%	102	11.9%	116	13.5%	88	10.3%	67		7.8%	
People who are very unhappy with their physical appearance should consider cosmetic surgery as one option	138	16.1%	156	18.2%	192	22.4%	106	12.4%	130	15.2%	78	9.1%	57		6.7%	
If cosmetic surgery can make someone happier with the way they look, then they should try it	144	16.8%	186	21.7%	143	16.7%	118	13.8%	122	14.2%	75	8.8%	69		8.1%	
If I could have a surgical procedure done for free, I would consider trying cosmetic surgery	208	24.3%	117	13.7%	200	23.3%	90	10.5%	94	11.0%	67	7.8%	81		9.5%	
If I knew there would be no negative side effects or pain, I would like to try cosmetic surgery	160	18.7%	178	20.8%	140	16.3%	102	11.9%	99	11.6%	77	9.0%	101		11.8%	
I have sometimes thought about having cosmetic surgery	185	21.6%	127	14.8%	193	22.5%	69	8.1%	114	13.3%	84	9.8%	85		9.9%	
I would seriously consider having cosmetic surgery if my partner thought it was a good idea	222	25.9%	194	22.6%	142	16.6%	98	11.4%	81	9.5%	55	6.4%	65		7.6%	
I would never have any kind of plastic surgery	94	11.0%	61	7.1%	98	11.4%	128	14.9%	197	23.0%	135	15.8%	144		16.8%	
I would think about having cosmetic surgery in order to keep looking young	215	25.1%	173	20.2%	155	18.1%	100	11.7%	94	11.0%	58	6.8%	62		7.2%	
If it would benefit my career, I would think about having plastic surgery	194	22.6%	124	14.5%	203	23.7%	100	11.7%	107	12.5%	71	8.3%	58		6.8%	
I would seriously consider having cosmetic surgery if I thought my partner would find me more attractive	246	28.7%	175	20.4%	132	15.4%	96	11.2%	98	11.4%	61	7.1%	49		5.7%	
Cosmetic surgery can be a big benefit to people's self-image	90	10.5%	114	13.3%	195	22.8%	98	11.4%	129	15.1%	112	13.1%	119		13.9%	
If a simple cosmetic surgery procedure would make me more attractive to others, I would think about trying it	227	26.5%	176	20.5%	153	17.9%	86	10.0%	98	11.4%	68	7.9%	49		5.7%	

Table 3 describes the acceptance of cosmetic surgery by domain and overall among study participants in Saudi Arabia. The highest score in percentage was for the interpersonal subscale (18.7±7.9; 53.4%), followed by the consider subscale (18.2±7.2; 52%) and social subscale (15.5±7.9; 44.3%). The overall mean score was 52.4±21.1; 49.9%.

**Table 3:** Description of the acceptance of cosmetic surgery by the domain and overall among study participants in Saudi Arabia

Acceptance domains	Minimum	Maximum	Mean	SD	% of the total score
Intrapersonal score	5	35	18.7	7.9	53.4%
Social score	5	35	15.5	7.9	44.3%
Consider score	5	35	18.2	7.2	52.0%
Overall score	15	105	52.4	21.1	49.9%

**Figure 1:**
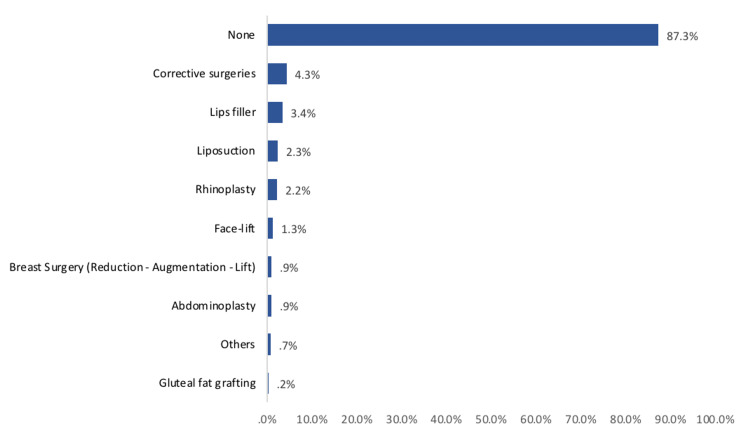
History of undergoing cosmetic surgery among study participants in Saudi Arabia

The history of undergoing cosmetic surgery among study participants in Saudi Arabia is presented in Figure 1. The most reported cosmetic surgery undergone was corrective surgeries (4.3%), followed by lip fillers (3.4%), liposuction (2.3%), rhinoplasty (2.2%), and facelift (1.3%) while 87.3% had never undergone any cosmetic surgery.

**Figure 2:**
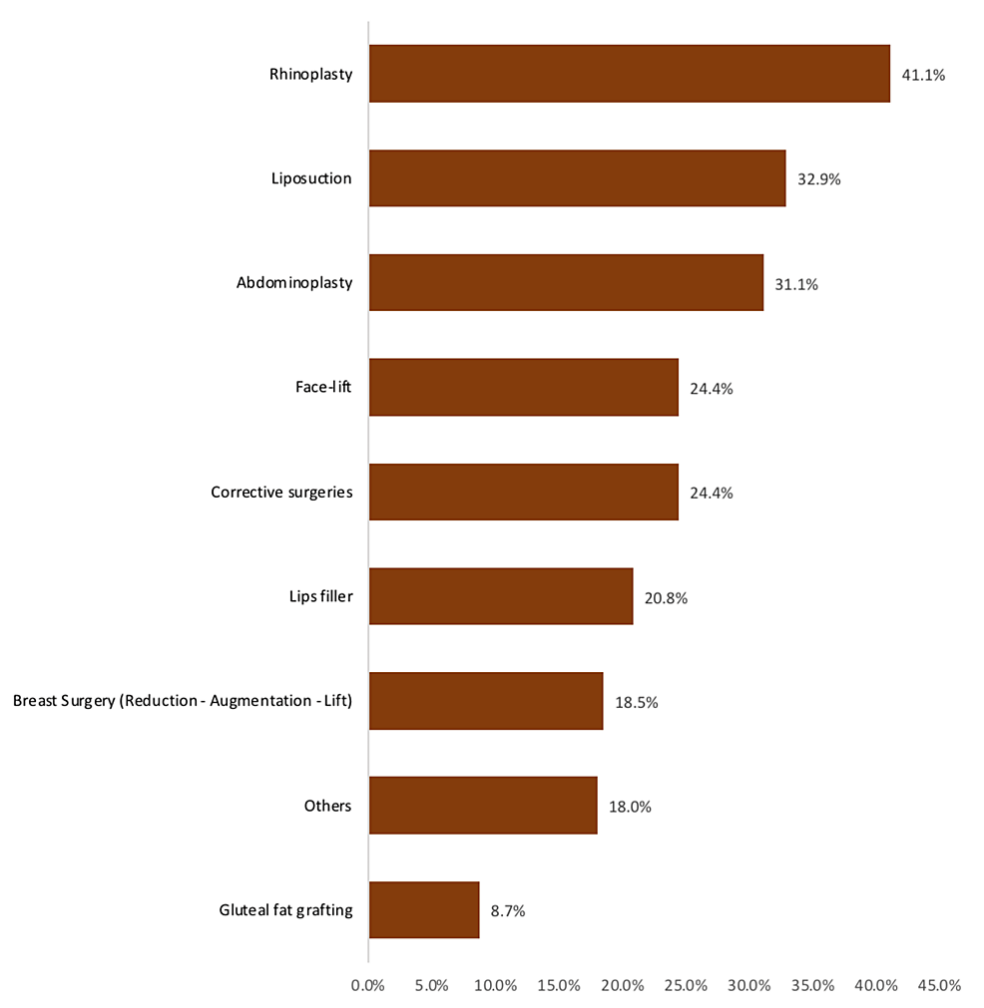
The type of procedure study participants would like to have in Saudi Arabia

However, Figure 2 shows the type of procedure participants would like to have among the study participants in Saudi Arabia. The most reported was rhinoplasty (41.1%), followed by liposuction (32.9%), abdominoplasty (31.1%), facelift (24.4%), corrective surgeries (24.4%), and lip fillers (20.8%) while the least intended was gluteal flat grafting (8.7%).

Finally, Table 4 measures the association between participants’ overall acceptance scores with their personal data. The overall mean acceptance core was significantly higher among Western region participants (56.9 ± 20.3) than among Northern region participants (40.1± 8.8) with a recorded statistical significance (P=.039). Also, female participants had a mean acceptance score of 55 ± 21.5 versus 45.3 18.3 for male participants (P=.001). Additionally, the overall mean score for participants with a low-income level was 55.4 ± 21.0 compared to 49.8 20.4 of those with a monthly income of 10000-20000 SR (P=.040).

**Table 4:** Association between participants' overall acceptance score with their personal data

Factors	Overall score		p-value
	Mean	SD	
Region			.039*
Central region	55.0	19.6	
Eastern region	54.2	23.6	
Northern region	40.1	8.8	
Southern region	49.1	20.4	
Western region	56.9	20.3	
Age in years			.207
< 19	49.0	23.2	
19-25	54.2	20.1	
26-35	52.3	21.0	
36-45	49.2	21.7	
46-55	52.9	23.3	
> 55	51.1	21.4	
Gender			.001*^$^
Male	45.3	18.3	
Female	55.0	21.5	
Marital status			.120
Single	52.0	20.6	
Married	53.5	22.0	
Divorced/widow	46.0	16.8	
Education			.587
Below secondary	51.8	22.5	
Diplomat	57.1	23.8	
University/above	52.4	20.8	
Job			.351
Not working	54.4	21.9	
Student	52.7	21.3	
Employee	51.3	20.6	
Monthly income			.040*
< 5000 SR	55.4	21.0	
5000-10000 SR	52.2	19.9	
10000-20000 SR	49.8	20.4	
> 20000 SR	53.4	24.1	

## Discussion

Over the past two decades, there has been a global increase in the number of cosmetic procedures performed, with a rate of 169% [5]. This rise in the popularity of cosmetic surgery denotes a profound shift in the way people view and accept cosmetic surgery [16]. Therefore, the purpose of the current study was to assess the perception and acceptance of the Saudi population toward aesthetic procedures and to understand the factors influencing their level of acceptance. To achieve this goal, a cross-sectional study using an online questionnaire was carried out. According to our data, women make up 73.5% of the study sample while men make up only 26.5%. Age-wise, 65.4% of our participants were in the 19- to 35-year-old range. Our findings extend previous efforts with the ACSS) and demonstrate how sociodemographic information can be used to explain the variation in participants' overall level of acceptance.

Our study's overall ACSS score was 52.4, which was lower in comparison to recent studies conducted in Saudi Arabia. Morait et al. reported a mean ACSS score of 60.11±25.11 among 389 adults in Riyadh, with a mean age of 29 years old [14]. Additionally, Al Ghadeer et al. found a mean ACSS score of 58.2±20.5 among 1008 adults in the Eastern Province, with a mean age of 34 years old [17]. This enormous disparity between our results can be attributed to the difference in the mean age of the research participants, as it was reported that acceptance of cosmetic interventions is remarkably higher in older age groups [15,18]. However, it should be noted that in our study, the acceptability of cosmetic surgeries was not found to be significantly influenced by age. Similar conclusions were reached in previous studies done in South Korea and Italy [3,19].

Concerning overall ACSS score and gender, our results demonstrated that women had a considerably higher score compared to men. This was in line with other research showing that Saudi women are more likely than men to undergo cosmetic surgery in Saudi Arabia [14]. Similarly, studies that were conducted in Nigeria [20] and Serbia [16] reported that women were significantly more accepting of cosmetic surgeries. This gender disparity may be caused by the increased sociocultural pressure placed on women to meet the standards of physical beauty and attractiveness [8,20].

Our research showed that internal factors had a significant impact on Saudi Arabians' decision to undergo cosmetic surgery. Our result ties well with previous studies in America [15] and Italy [19] showing that the intrapersonal scale had the highest score followed by the consider scale. On the other hand, there was no discernible difference between internal and external motives in a study among Korean women [21]. Furthermore, Swami V et al. suggested that this disparity may be due to the cultural variations between societies that value individualism and those that emphasize collectivism [21]. Nevertheless, cosmetic surgery is one method that can be applied for a variety of goals. It can help one's body remain healthy over time, which meets their desire to delay the aging process; but it is also seen as a way to satisfy social expectations, most likely related to the adoption of common aesthetic standards [21].

This is one of the few studies that include participants from all the regions in Saudi Arabia. However, this study has several limitations. First, the use of online channels to distribute the survey might have resulted in a selection bias against people who were not able to access it at the time. Second, the sample wasn't dispersed evenly among the Kingdom’s regions. Consequently, results gained in such particular contexts might not be entirely representative of the entire population.

## Conclusions

According to the current study, Saudi women are more likely than Saudi men to have cosmetic surgery in Saudi Arabia. The greater sociocultural pressure on women to uphold ideals of physical attractiveness may be the root of this gender gap. Saudi Arabians' decisions to have cosmetic surgery were significantly influenced by internal considerations. Additionally, age did not have a major impact on the acceptance of cosmetic surgery. Additional research should be conducted to determine whether disparities exist regarding gender and culture.
